# Dairy products, calcium and prostate cancer risk

**DOI:** 10.1038/sj.bjc.6603475

**Published:** 2006-11-14

**Authors:** K A Koh, H D Sesso, R S Paffenbarger, I-M Lee

**Affiliations:** 1 Harvard University, 8 Garden Street, Cambridge, MA 02138, USA; 2 Department of Medicine, Division of Preventive Medicine, Brigham and Women's Hospital and Harvard Medical School, 900 Commonwealth, Avenue East, Boston, MA 02215, USA; 3 Department of Epidemiology, Harvard School of Public Health, 677 Huntington Avenue, Boston, MA 02115, USA; 4 Division of Epidemiology, Department of Health Research Policy, HRP Redwood Building, T213B, Stanford, CA 94305, USA

**Keywords:** calcium, diet, epidemiology, prostate cancer, supplements, vitamin D

## Abstract

In a prospective study of 10 011 men with 815 prostate cancer cases, despite plausible biological mechanisms, neither increasing intake levels of dairy products nor calcium from dairy products (*P* trend; 0.23 and 0.64, respectively), or calcium supplements was associated with prostate cancer risk (relative risk, 1.05; 95% confidence interval, 0.84–1.31).

Prostate cancer is the most commonly diagnosed non-skin cancer among men in North America, Europe, and parts of Africa, with the United States having the highest prostate cancer incidence in the world (Gronberg, 2003). Although its incidence is increasing steadily in almost all countries, few modifiable predictors for the disease have been identified (Gronberg, 2003). Well-established risk factors, such as age, family history, race, and country of residence, are not amenable to modification and have limited utility as primary prevention strategies.

Calcium intake, a modifiable dietary factor, has recently been proposed as a risk factor for prostate cancer. Evidence from *in vitro* and animal studies suggests that risk may be increased by higher levels of calcium, since these suppress 1,25-dihydroxyvitamin D_3_, the most active form of vitamin D_3_, that inhibits the proliferation of prostate cancer cells and promotes their differentiation (Schwartz *et al*, 1995, 1997; Giovannucci, 1998). However, relevant studies have yielded inconsistent results (Gao *et al*, 2005), although few studies have considered intake from calcium supplementation, that can provide higher doses than dietary intake. Given calcium's importance in the prevention of diseases such as osteoporosis, we conducted a study of the question in a large cohort of men.

## MATERIALS AND METHODS

### Subjects

The Harvard Alumni Health Study, initiated in the 1960s, is an ongoing cohort study of men who entered Harvard University, United States, as undergraduates between 1916 and 1950. Health information is obtained from subjects via questionnaires mailed at periodic intervals. The present study utilises data from a questionnaire mailed in 1988 that included detailed information on diet, and eligible subjects were 12 805 men who responded. We excluded men reporting cancer at baseline (*n*=1731) and those not providing information on dairy product intake (*n*=60). Of the remaining 11 014 men, we successfully followed 10 011 (90.9%) for the development of prostate cancer; these men represent the subjects for the present analysis.

### Assessment of calcium intake and other factors

The 1988 questionnaire assessed diet using an abbreviated version (23 food items) of a validated semiquantitative food frequency questionnaire (Willett *et al*, 1985, 1987). Men indicated their usual daily intake of seven different dairy products (whole milk, low fat milk, cream, ice cream, yogurt, cheese and butter) using pre- specified responses ranging from ‘almost never’ to ‘six or more times per day.’ On the original, longer version of the questionnaire, 13 dairy items were assessed. However, we believe our assessment of calcium intake using the seven items s reasonably valid. While we did not directly validate our 7-item questionnaire against the longer questionnaire, a previous study that assessed fewer items – milk (including whole, skim and milk with cereal), cheese and ice cream – was able to account for 82% of the variation in calcium consumption in male physicians (Chan *et al*, 2001).

We estimated calcium intake in mg day^−1^ based on the frequency of consumption of each dairy product and the calcium composition of specified portions of each dairy product, using data from the US Department of Agriculture (US). In addition to dairy products, we asked alumni about use of calcium supplements (occasional use, use of ordinary daily dose, or use of high daily dose).

From the 1988 questionnaire, we also obtained information on potential confounders, including weight, height, physical activity, cigarette smoking, alcohol intake, and paternal history of prostate cancer.

### Ascertainment of prostate cancer

Men reported physician-diagnosed prostate cancer on subsequent health questionnaires in 1993 and 1998. A previous validation study has shown that such self-reports were confirmed by attending physicians in 91% of cases (Lee *et al*, 1992). In addition, we obtained death certificates for men who died through 1998 to ascertain cases of fatal prostate cancer.

### Data analyses

In separate analyses, we considered intakes of (1) dairy products, (2) calcium from dairy products, and (3) calcium supplements, in relation to prostate cancer risk. For dairy products, we created a dairy score, representing servings of dairy products per day, by summing the frequency of consumption of the seven dairy products, and categorised men into approximate quartiles (Table 1). Similarly, we grouped men into approximate quartiles of calcium intake, in mg day^−1^, based on the frequency of consumption of each dairy product and the calcium composition of each dairy product (Table 2). For supplements, because only 8% of men reported using supplemental calcium (with most taking an ordinary daily dose), we grouped men as non-users or users. We used Cox proportional hazards regression models to estimate the relative risks (RR) and 95% confidence intervals for developing prostate cancer associated with dairy and calcium intakes. Initially, we adjusted for age (in continuous years) only, but later for the following potential confounders: body mass index (<22.5, 22.5–<25.0, 25.0–<27.5, or ⩾27.5 kg m^−2^), physical activity (<1000, 1000–2499, or ⩾2500 kcal week^−1^), smoking (never, past, or current), total energy intake (in quartiles), alcohol intake (none, 1–3, 4–6, or ⩾seven drinks week^−1^), red meat intake (⩽three servings month, 1–2 servings week, or ⩾three servings week^−1^), vegetable intake (⩽six servings week, 1–2 servings day, or ⩾three servings day^−1^), and paternal history of prostate cancer (no or yes). We repeated the above analyses, examining only fatal prostate cancer, owing to the suggestion from some previous studies that the association may be stronger with advanced or fatal prostate cancer (Giovannucci *et al*, 1998; Schuurman *et al*, 1999; Chan *et al*, 2001; Rodriguez *et al*, 2003).

## RESULTS

Among the 10 011 men, the mean age at baseline in 1988 was 67 years. Table 1 shows the baseline characteristics of subjects according to dairy product intake. Men in the highest quartile of dairy consumption tended to be older, were more likely to smoke, and were less likely to be overweight. They also consumed more red meat and vegetables, and were more likely to have a paternal history of prostate cancer. There were no clear trends for physical activity and alcohol consumption.

During follow-up through 1998, 815 incident cases of prostate cancer developed, of which 99 cases were fatal. Table 2 shows no significant relation between higher intakes of dairy products and risk of prostate cancer, whether in age- or multivariate-adjusted analyses (*P* trend=0.16 and 0.23, respectively). In multivariate analyses, men in the highest quartile had an RR of prostate cancer of 1.11 (95% confidence interval, 0.85–1.46), compared with the lowest quartile. When individual dairy products were examined, there were also no significant associations (data not shown).

For calcium intake, Table 2 shows that levels of intake were not significantly associated with prostate cancer risk, either in age- or multivariate-adjusted analyses (*P* trend=0.93 and 0.64, respectively). Calcium supplement use was also not significantly related to risk (multivariate RR=1.05 (0.84–1.31)).

When we repeated the above analyses, examining only fatal (instead of all) prostate cancer, we continued to observe no significant associations between dairy product or calcium intakes and risk of fatal prostate cancer (*P* trend=0.64 and 0.52, respectively; Table 3).

## DISCUSSION

This large study does not support the hypothesis that higher intakes of calcium are associated with an increased risk of prostate cancer. We observed no significant associations between the intake of dairy products, dairy calcium, or calcium supplements and risk of either all or fatal prostate cancer. It is important to clarify the subject because calcium intake clearly is beneficial for other diseases, such as osteoporosis (Prentice, 2002). The UK Department of Health recommends that adults consume 700 mg day^−1^ of calcium (Prentice, 2002); in the United States, the Institute of Medicine recommends 1000–1200 mg day^−1^ (Prentice, 2002).

Although we did not observe any significant associations, it appears biologically plausible for higher calcium intakes to increase prostate cancer risk. High circulating levels of calcium suppress 1,25-dihydroxyvitamin D_3_, the most active form of vitamin D. *In vitro*, 1,25-dihydroxyvitamin D_3_ inhibits the growth of cancerous prostate epithelial cells and promotes their differentiation (Schwartz *et al*, 1995, 1997; Giovannucci, 1998). Animal studies also have shown that administration of 1,25-dihydroxyvitamin D_3_ or its analogues reduces the growth of prostate gland tumours (Lucia *et al*, 1995). Additionally, higher intakes of milk and calcium may increase insulin-like growth factor-1 (IGF-1) (Gunnell *et al*, 2003), which may be associated with increased prostate cancer risk (Renehan *et al*, 2004).

Findings from previous studies have been conflicting. While some large cohort studies (with >500 cases) have reported significant positive associations between dairy products and/or calcium intake and risk (Giovannucci *et al*, 1998; Chan *et al*, 2001), others have not (Schuurman *et al*, 1999; Michaud *et al*, 2001; Rodriguez *et al*, 2003). A recent clinical trial of calcium supplementation (1200 mg day^−1^) in the prevention of colorectal adenomas found no significant increase in prostate cancer (70 cases) risk with supplementation in secondary analyses, and even a suggestion of a protective effect (Baron *et al*, 2005). A 2005 meta-analysis of prospective cohort studies reported increased RR of 1.11 (95% confidence interval, 1.00–1.22) and 1.39 (1.09–1.77) for all prostate cancer among men with the highest intakes of dairy products and calcium, respectively, and stronger associations for advanced prostate cancers (RR=1.33 and 1.46, respectively) (Gao *et al*, 2005). However, with the inclusion of a 2006 Australian study that found no association with calcium intake (Severi *et al*, 2006), the findings for all prostate cancer were weakened (RR=1.09 (1.00–1.20) for dairy products; 1.32 (1.05–1.67) for calcium) (Severi *et al*, 2006). The present study agrees with the Australian data, in showing no significant relation between calcium intake and risk.

Strengths of the present study include its prospective design, large number of cases (including fatal cases), and high rates of follow-up. Additionally, dietary information was ascertained using a validated food frequency questionnaire, and information was available on several potential confounders. The study also included many older men (baseline age 67 years), who are at greater risk for prostate cancer (Gronberg, 2003).

One potential limitation is that the calcium intake may have been too low in the present study. The highest category of calcium intake from dairy products was ⩾600 mg day^−1^ (median 849 mg day^−1^; interquartile range 710–1416 mg day^−1^). Thus, many men would have had intakes below US recommendations, and several would have had intakes falling below UK recommendations (Prentice, 2002). However, a previous large study did observe a significantly increased risk at ⩾600 mg day^−1^ calcium intake (Chan *et al*, 2001), making it less likely that the range of intakes was responsible for our null findings. Further, since we relied on self-reported diet, random misclassification may have occurred, biasing findings to the null. However, the large number of cases offers some assurance that an association of moderate magnitude, as has been reported in previous studies (Giovannucci *et al*, 1998), would not have been missed. Finally, we were specifically interested in advanced prostate cancer, but did not have information on the stage or grade of prostate cancers diagnosed in the present study. However, we were able to ascertain fatal prostate cancers, which would reasonably reflect advanced disease.

In conclusion, this study does not support the biologically plausible hypothesis that a high calcium intake increases prostate cancer risk. Further studies should include other populations, such as Blacks, in whom prostate cancer rates are higher (Hayes *et al*, 1999; Gronberg, 2003), to extend the generalisability of the present findings.

## Figures and Tables

**Table 1:** Baseline characteristics of men in 1988 according to dairy product intake, Harvard Alumni Health Study

	**Dairy product intake (servings/day)^a^**			
**Characteristic**	**0–<1.25 (*n*=2460)**	**1.25–<2.00 (*n*=2537)**	**2.00–<3.25 (*n*=2416)**	**⩾3.25 (*n*=2598)**
Mean age (s.d.) (years)	66.0 (7.5)	66.9 (7.9)	67.3 (8.0)	68.7 (8.4)
Cigarette smoking (%)	7.6	7.6	6.7	10.1
Overweight or obese (%)^a^	46.0	43.4	42.6	39.4
Physical activity ⩾1000 kcal/week (%)^b^	67.3	70.2	69.3	66.2
Alcohol intake (%)	71.4	73.0	73.7	70.4
Red meat intake ⩾3 servings/week (%)	21.2	25.3	33.4	38.7
Vegetable intake ⩾3 servings/day (%)	14.2	12.6	13.7	18.6
Paternal history of prostate cancer (%)	3.2	3.5	4.8	4.2

a Body mass index ⩾25 kg m^−2^.

b Sufficient to meet recommended level of physical activity.

**Table 2:** RR of total prostate cancer according to dairy product and calcium intake, Harvard Alumni Health Study

	**Age-adjusted RR (95% confidence interval)**	**Multivariate RR^a^ (95% confidence interval)**
*Dairy product intake (servings/day)* b		
0–<1.25 (189 cases)	1.00 (referent)	1.00 (referent)
1.25–<2.00 (183 cases)	0.91 (0.74–1.12)	0.91 (0.71–1.15)
2.00–<3.25 (220 cases)	1.14 (0.90–1.39)	1.11 (0.87–1.42)
⩾3.25 (223 cases)	1.07 (0.88–1.31)	1.11 (0.85–1.46)
	*P* trend=0.16	*P* trend=0.23
		
*Dairy calcium intake (mg/day)* b		
0–199 (209 cases)	1.00 (referent)	1.00 (referent)
200–449 (167 cases)	0.83 (0.68–1.02)	0.81 (0.64–1.02)
450–599 (238 cases)	0.97 (0.81–1.17)	0.91 (0.73–1.14)
⩾600^c^ (201 cases)	0.96 (0.79–1.17)	0.91 (0.70–1.18)
	*P* trend=0.93	*P* trend=0.64

RR=relative risks.

a Adjusted for age; smoking; body mass index; physical activity; intakes of alcohol, red meat and vegetables; total caloric intake; and paternal history of prostate cancer.

b Summed from intakes of milk, low-fat milk, cream, yogurt, cheese, ice cream, and butter.

c Median 849 mg day^−1^; interquartile range 710–1416 mg day^−1^.

**Table 3:** RR of fatal prostate cancer according to dairy product and calcium intake, Harvard Alumni Health Study

	**Age-adjusted RR (95% confidence interval)**	**Multivariate RR^a^ (95% confidence interval)**
*Dairy product intake (servings/day)* b		
0–<1.25 (25 cases)	1.00 (referent)	1.00 (referent)
1.25–<2.00 (21 cases)	0.74 (0.41–1.32)	0.54 (0.25–1.21)
2.00–<3.25 (23 cases)	0.81 (0.46–1.43)	0.71 (0.33–1.53)
⩾3.25 (30 cases)	0.91 (0.53–1.56)	1.12 (0.51–2.47)
	*P* trend=0.88	*P* trend=0.64
		
*Dairy calcium intake (mg/day)* b		
0–199 (30 cases)	1.00 (referent)	1.00 (referent)
200–449 (21 cases)	0.72 (0.41–1.26)	0.57 (0.27–1.19)
450–599 (23 cases)	0.63 (0.37–1.09)	0.60 (0.29–1.22)
⩾600^c^ (25 cases)	0.74 (0.44–1.26)	0.81 (0.38–1.71)
	*P* trend=0.22	*P* trend=0.52

RR=relative risks.

a Adjusted for age; smoking; body mass index; physical activity; intakes of alcohol, red meat and vegetables; total caloric intake; and paternal history of prostate cancer.

b Summed from intakes of milk, low-fat milk, cream, yogurt, cheese, ice cream, and butter.

c Median 849 mg day^−1^; interquartile range 710–1416 mg day^−1^.
